# Causes of death in Prader-Willi syndrome: lessons from 11 years’ experience of a national reference center

**DOI:** 10.1186/s13023-019-1214-2

**Published:** 2019-11-04

**Authors:** Dibia Liz Pacoricona Alfaro, Perrine Lemoine, Virginie Ehlinger, Catherine Molinas, Gwénaëlle Diene, Marion Valette, Graziella Pinto, Muriel Coupaye, Christine Poitou-Bernert, Denise Thuilleaux, Catherine Arnaud, Maithé Tauber

**Affiliations:** 1 UMR 1027 Inserm- Paul Sabatier University, Toulouse, France; 2 Endocrinology, Obesity, Bone Diseases, Genetics and Gynecology Unit, Children’s Hospital, University Hospital Center of Toulouse, Toulouse, France; 3 French National Reference Center for Prader-Willi Syndrome, Children’s Hospital, University Hospital Center of Toulouse, 330, avenue de Grande-Bretagne - TSA 40031, 31059 Toulouse cedex 9, France; 4 Centre de Physiopathologie de Toulouse-Purpan, UMR 5282 CNRS, UMR 1043 Inserm, Paul Sabatier University, Toulouse, France; 5 Pediatric Endocrinology, Diabetology and Gynecology Department, Assistance-Publique Hôpitaux de Paris (AP-HP), Necker Children’s University Hospital, Paris, France; 6 French National Reference Center for Prader-Willi Syndrome, Nutrition Department, Assistance-Publique Hôpitaux de Paris (AP-HP), Pitié-Salpêtrière Hospital, Paris, France; 7 Nutriomics team, Sorbonne University, UPMC University Paris 06, Inserm, Paris, France; 8 French National Reference Center for Prader-Willi Syndrome, Prader-Willi Unit, Assistance Publique Hôpitaux de Paris (AP-HP), Marine Hendaye Hospital, Hendaye, France; 9 Unité de Soutien Méthodologique à la Recherche, University Hospital Center of Toulouse, Toulouse, France

**Keywords:** Prader-Willi syndrome, Epidemiology, Mortality, Respiratory complications, Sudden death

## Abstract

**Background:**

In the last 20 years, substantial improvements have been made in the diagnosis, treatment and management of patients with Prader-Willi syndrome (PWS). Few data on causes of death are available since those improvements were made. Our study assessed the causes of death among French patients with PWS over the first 11 years of experience of the nationwide French Reference Center for PWS (FRC-PWS).

**Methods:**

Our study relied on two sources of mortality information at national level between 2004 and 2014: The French Epidemiological Centre for the Medical Causes of Death (CépiDc) Registry and the FRC-PWS database. Causes of death were classified into seven categories: respiratory, cardiovascular, gastrointestinal, severe infection, sudden death, other causes, and unknown. Descriptive statistics were calculated separately for children (< 18 years-old) and adults (≥18 years-old).

**Results:**

One hundred and four deaths were identified in France from 2004 to 2014. The median age at death was 30 years, ranging from less than 1 month to 58 years. Seventeen deaths occurred in patients under 18 years, with 70% of them in children under 2 years. Respiratory causes accounted for more than 50% of the deaths in patients with PWS in both children and adults. Both cause and age of death did not significantly differ according to gender or genetic subtype.

**Conclusions:**

Patients with PWS die prematurely due to a respiratory cause in most cases at all ages. In those adult patients with data on obesity, 98% were reported to be obese.

## Introduction

Prader-Willi syndrome (PWS) is a rare and complex neurodevelopmental genetic disease comprising multiple cognitive, behavioral and endocrine abnormalities. Birth prevalence has been estimated at 1/20,000 to 1/30,000 births [1–3]. The natural history of the disease has been described as having successive phases starting during the last trimester of pregnancy [4]. After birth, this syndrome is characterized by neonatal hypotonia and poor sucking with feeding difficulties that lead to nasogastric tube feeding in most cases and possible failure to thrive in early infancy [5]. Subsequently, in the absence of early care, spontaneous excessive weight gain occurs at about 3 years, with no increase in food intake, followed by typically severe obesity with hyperphagia and a satiety deficit [6].

Greater awareness among neonatologists and the availability of genetic testing for PWS have led to early diagnosis, now in the first weeks of life in most countries [7–9]. Early identification and multidisciplinary care help to prevent obesity, and detect and treat comorbidities [7]. In addition, growth hormone (GH) treatment was labeled in 2000 as an orphan drug for PWS and contributes to growth and healthier body composition by improving the lean mass/fat mass ratio [10, 11]. GH treatment has also been shown to improve motor and cognitive development, enhancing the quality of life of patients with PWS [12].

Despite the vast progress in the last 20 years, we have little evidence of a significant impact on the mortality rate among patients with PWS, yet it has been estimated to be three times higher than for the general population (3% per year) [13]. The literature has identified the complications of severe obesity as the main cause of death in adults [14–19], whereas death in children is mostly due to respiratory illness [15, 20, 21]. Nevertheless, most of these data have been drawn from case studies or small patient series [17, 20, 22–24]. Only few studies were based on substantially higher numbers of patients. In a previous work [21], we reviewed publications and case reports of child and adolescent deaths during the period 1980–2007. We identified 64 cases of childhood death, described their causes and showed that the major cause of death in PWS children was respiratory diseases, with no difference between those receiving and not receiving GH treatment. Otherwise, since 1999, the US Prader-Willi Association has developed a bereavement support program for families. With the collaboration of American PWS experts, they were able to collect retrospective and prospective data on the causes of death. An article published in 2007 partially described the deaths of 152 patients from the US Prader-Willi Association Registry, with a focus on choking-related deaths and scarce information about other causes [25]. A recent article from Butler and colleagues identified causes of death among 312 patients with PWS, with respiratory failure being the most common cause [18]. Their article covered the causes of death over a 40-year period, which explains the high rate of missing data and the heterogeneity in the organization and type of care. Nevertheless, these data allowed Manzardo and colleagues [26] to explore survival trends and to highlight differences between patients who died before and after 2000. With the most recent data from the US Prader-Willi Association Registry (2004–2014), new analyses were published [19]. A total of 114 deaths across the United States were identified and compared with the living patients with PWS present in the registry, although the causes of death were not detailed in this article.

Along with the substantial modifications in the management of patients with PWS since the 2000s, public health policies have also been developed. In France, the National Plan for Rare Disease, launched in 2004 by the French Ministry of Health, structured the care of people with rare disorders through the designation of Reference Centers associating scientific expertise and medical competences. The French Reference Center for PWS was labeled in 2004 and has three sites: the Children’s Hospital (Toulouse), the Pitié-Salpêtrière Hospital (Paris) and the Marine Hospital (Hendaye). The aim of the Reference Center is to optimize access to care for patients of all ages by improving knowledge on the disease and good practices, and by training and organizing hospitals throughout the country with expertise in PWS care designated as Competence Centers. In addition, the Reference Center conducts research on PWS, which encompasses basic research, clinical trials and epidemiological surveillance. For the purpose of research, a national database was implemented. The database provides a large amount of information resulting from more than 10 years of experience of the Reference Center and this represents a unique opportunity for detailed and comprehensive epidemiological studies. The current study aimed to describe the causes of death among patients with PWS over the first 11 years of the Reference Center’s experience, from 2004 to 2014.

## Methods

This retrospective observational study is based on data from two sources of national mortality information: the French Reference Center for PWS (FRC-PWS) database and the French Epidemiological Center for the Medical Causes of Death (CépiDc) Registry.

The FRC-PWS database was constructed to register PWS cases and study the cohort of patients followed by the Reference Center [27, 28]. The database includes clinical, psychological and socio-demographic information. Data collection was performed at the time of inclusion (baseline information) and then at annual follow-ups. The questionnaire was completed by the patient’s physician based on a thorough medical history and physical examination. The family completed psychological and quality of life questionnaires. At each follow-up visit, socio-family characteristics were actualized, if modified and clinical data was updated. All questionnaires were centralized by the Reference Center in Toulouse, responsible for data entry. The deaths of patients with PWS were also recorded in the FRC-PWS database. To verify that we had access to all the cases of death and their causes, and to obtain additional information where needed, the 20 hospitals designated as Competence Centers for PWS and the French Prader-Willi Association were contacted. Notifications and causes of death were collected and matched with the FRC-PWS database. Genetic laboratories that perform routine PWS diagnosis in France were contacted to ascertain genetic subtypes.

CépiDc registers all cases of death in France and manages the non-nominative medical part which contains the causes of death. We thus had access to all cases of death of patients with a PWS diagnosis (identified with ICD diagnosis code Q87.1: Congenital malformation syndromes predominantly associated with short stature) between 2004 and 2014. All patients with a PWS diagnosis mentioned on their death certificate were selected for the study.

The data from the FRC-PWS and the CépiDc registry were matched based on gender, date of birth and year of death. The matching was performed without difficulty since we did not find two patients of the same gender born the same day and dead the same year.

The FRC-PWS database was presented to all patients with Prader-Willi syndrome by their physician during a medical consultation at the Reference Center. Parents and patients were informed that they have the right to refuse to participate and, even after beginning data collection, may refuse to provide any given information, and may choose to request the complete removal of their data from the database. Before collecting data of a patient into the FRC-PWS database, we obtained written consent from all parents or legal guardians. The FRC-PWS database has the approval of the French Commission for Data Protection and Liberties (CNIL), which allows access to the CépiDc data for research purposes. The data obtained via the CépiDC were anonymized.

Primary causes of death were classified by a PWS expert (Pr Tauber) into seven categories: respiratory, cardiovascular, gastrointestinal, severe infection, sudden death, other causes, and unknown. Respiratory causes were subdivided into two groups: respiratory failures and respiratory infections. Cardiovascular causes were sub-classified into cardiac failures, pulmonary embolisms or other cardiac causes. Gastrointestinal causes were subdivided into acute gastroenteritis and occlusion. Last, sub-classifications of severe infections were sepsis and other infectious causes. For patients only retrieved by the CépiDC recorded as “cardiorespiratory arrest” without further information, we categorized the death as sudden. For most of the patients registered in the FRC-PWS database, information about genetic subtype, GH treatment and the data on body mass index (BMI, the more recent data was considered) was available.

Descriptive statistics are expressed as medians (minimum-maximum), means (± standard deviation) and percentages as appropriate. Percentages for each variable were referred to the number of patients for whom the information was available. Data were split between two groups: children (age at death < 18 years old) and adults (age at death ≥18 years old). Primary causes of death were described and compared according to patient characteristics (gender, age at death and genetic subtype) using the Chi^2^ test or Fisher’s exact test in the case of small expected values, or the Mann-Whitney rank sum test, as appropriate. A *p*-value of 0.05 or less was set as statistically significant for all statistical tests. In the case of significant results, post hoc analyses were applied with a Bonferroni correction of the level of significance to determine which specific pairs of group means showed differences.

All analyses were performed using STATA v14.0.

## Results

### Patient characteristics

One hundred and four deaths of patients with PWS were identified in France from 2004 to 2014. The patients were born between 1951 and 2013.The number of deaths per year varied from year to year, ranging from 5 to 15. The median number of deaths per year was 9.

Forty-two cases of death (40.4%) were reported by both sources of information. Forty-six cases of death were reported only by the CépiDC. Sixteen cases of death were identified by the FRC-PWS but not recorded by the CépiDC. Age and causes of death did not differ between patients from both sources of information and those registered only from CépiDC registry or identified only by the FRC-PWS (data not shown). A total of fifty-eight patients were registered in the FRC-PWS database. Among them, 10 were < 18 years old. Thus, most of the cases of child death were known to the Reference Center and/or a Competence Center (10/17, 58.8%). The proportion of adults registered in the FRC-PWS database rose to 55.2%. Among patients born from the most recent generations (≥1990), 70.8% were registered in the FRC-PWS database. Only half of the patients (51.2%) born from older generations (1951–1989) were included in it.

**Fig. 1:**
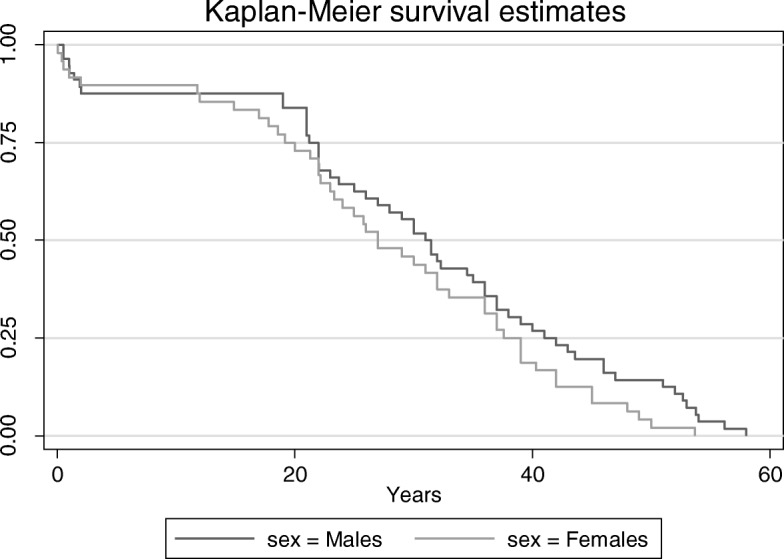
Kaplan-Meier plot of survival probability. Plot of death in Prader-Willi Syndrome by gender over the period 2004–2014 in France

In our study population, median age at death was 30 years (ranging from less than 1 month to 58 years). Seventeen deaths occurred in patients < 18 years (16%). We found no significant differences in the age at death between males and females. Figure 1 shows the estimated survival function (Kaplan-Meier method) by gender. Patient characteristics according to age group are presented in Table 1.

**Table 1:** Patient characteristics

	Children < 18 years old (*n* = 17)	Adults ***≥*** 18 years old (*n* = 87)	Total (*n* = 104)
*Age at death, median (min;max)*	1.4 (0.1;17.8)	32.0 (18.6;58.0)	30 (0.1;58.0)
*Gender, n (%)*	*Information available for 100% of patients*		
Male	7 (41%)	49 (56%)	56 (54%)
Female	10 (59%)	38 (44%)	48 (46%)
*Genetic subtype, n (%)*	*Information available for 37% of patients*		
Deletion	7 (70%)	18 (64%)	25 (66%)
Uniparental maternal disomy	2 (20%)	7 (25%)	9 (24%)
Imprinting defect	1 (10%)	3 (11%)	4 (10%)
*Growth hormone treatment, n (%)*	*Information available for 34% of patients*		
No	5 (45%)	16 (67%)	21 (60%)
Yes	6 (55%)	8 (33%)	14 (40%)
*Obesity, n (%)*	*Information available for 49% of patients*		
No	6 (75%)	1 (2%)	7 (14%)
Yes	2 (25%)	42 (98%)	44 (86%)

Among children, most deaths occurred in the first 2 years of life. Eight cases of death were documented among children ≤1 year. For children from > 1 to ≤2 years, 4 cases of death were reported. No cases were reported in children from > 2 to ≤11 years. Adolescents (> 11 to < 18 years old) with PWS accounted for 5 cases of death.

### Primary cause of death

Respiratory causes accounted for more than 50% of the deaths. Respiratory failure was reported for 42 patients. Respiratory infection was the cause of death for 13 patients including 4 children.

A cardiovascular origin was listed as the primary cause of death for 15 patients. Cardiac failure and pulmonary embolism caused 8 and 4 deaths, respectively. Three other deaths were due to aneurysm, cardiac tamponade and viral myocarditis.

Four deaths were due to gastrointestinal causes: a 2-year-old boy who died of acute gastroenteritis and 3 gastrointestinal occlusions. With respect to the latter three, they were all young adults: a 21-year-old woman who died of an acute abdominal syndrome due to a functional intestinal obstruction, a 19-year-old man suffered a gastric perforation after an obstruction and a 32-year-old woman who died of peritonitis as a complication of a small bowel volvulus.

Severe infections leading to death were: 1 pneumococcal meningitis, 1 necrotizing infection of the abdominal wall and 2 sepsis. The origin of sepsis was unknown for both patients. For one of them, as the data came from the CépiDC, we could not obtain more precise information. The other patient was a 39-year-old man with multiple comorbidities (chronic renal failure, hypertrophy of the left ventricle with arrhythmia and complicated diabetes) for whom precise information on the origin of the sepsis could not be obtained.

Sudden deaths were reported for 18 patients. Eight of them were only identified through the CépiDC registry, 3 were only registered by the FRC-PWS database and 7 in both sources of information. For these last 7 cases, we considered the cause of death as sudden when in one of the sources the cause was coded as “sudden death” and in the other the information was “cardiorespiratory arrest” without more details or “unknown”; or when in one of the sources the cause was “cardiorespiratory arrest” and in the other “unknown”. Three patients died from miscellaneous causes. For one patient underlying condition was an end-stage renal failure. Another patient had a fatal neurologic event, status epilepticus. For the third patient, the cause of death was reported as “strangled” with no further detail in the CépiDC data. Since the patient was not registered in the FRC-PWS, the context of death remains unclear. For 5 patients, the cause of death was unknown in both or one of the sources of information (if the patient was reported only via one of the sources). In the period analyzed we did not register deaths due to cancer or leukemia.

The causes of death differed between children and adults (*p* = 0.017) (Table 2). A post-hoc analysis using Chi^2^ test revealed a significant difference between children and adults for death caused by respiratory failure (*p* = 0.009). No significant differences were found between the two groups for death due to respiratory infection or sudden death.

**Table 2:** Causes of death among patients with Prader-Willi syndrome by age

Causes of death	Children < 18 years old (*n* = 17), *n (%)*	Adults ***≥*** 18 years old (*n* = 87), *n (%)*	Total (*n* = 104)
Respiratory cause			55
*Respiratory failure*^*a*^	2 (12%)	40 (46%)	
*Respiratory infection*	4 (23%)	9 (10%)	
Cardiovascular cause			15
*Cardiac failure*	2 (12%)	6 (8%)	
*Pulmonary embolism*	0	4 (5%)	
*Other cardiovascular causes*	2 (12%)	1 (1%)	
Gastrointestinal cause			4
*Occlusion*	0	3 (3%)	
*Acute gastroenteritis*	1 (6%)	0	
Severe infection			4
*Sepsis*	0	2 (2%)	
*Other infectious cause*	1 (6%)	1 (1%)	
Sudden death	4 (23%)	14 (16%)	18
Other causes	0	3 (3%)	3
Unknown cause	1 (6%)	4 (5%)	5

^a^Significant difference between children and adults, *p* = 0.009

The primary causes of death among children in the first year of life were: respiratory infection (*n* = 2), cardiac failure (*n* = 1), cardiac tamponade (*n* = 1), pneumococcal meningitis (*n* = 1), and sudden death (*n* = 2). For one child, the cause of death was unknown.

Among children aged > 1 to ≤2 years, 2 deaths were due to respiratory causes (one respiratory failure, one respiratory infection). The 2 remaining cases were due to infectious diseases: viral myocarditis and acute gastroenteritis.

The common point for the 3 children under the age of 2 years who died after a respiratory infection was that they all had symptoms of a viral upper respiratory infection without fever in the days leading up to the fatal outcome. Those children were registered in the FRC-PWS database. Post-mortem microbiological analysis revealed a respiratory syncytial virus infection in the oldest.

The primary causes of death among adolescents with PWS were: respiratory failure (*n* = 1), respiratory influenza infection (*n* = 1), cardiac failure (*n* = 1) and sudden death (*n* = 2).

### Case of death according to patient characterisitics

No statistically significant differences in gender were found regarding the causes of death.

The causes of death among patients with an identified genetic subtype were not significantly different between patients with a deletion and patients with uniparental maternal disomy or an imprinting defect. Between these two groups, no statistically significant differences were observed regarding the median age of death. When children and adults were analyzed separately, no differences were observed for the causes of death or the distribution of the age at death. Among patients with a deletion, no significant gender differences were found regarding cause of death or age at death.

Fourteen patients had been treated with GH in the past or were being treated with it at the time of death. Median age at death among those patients was 19.6 years old (1.9 to 39 years).

Of the 6 children treated with GH, 4 were under GH treatment at the time of death. A 17.8-year-old adolescent had no GH treatment at the time of death caused by respiratory infection. For a 2-year-old patient, no information about GH treatment at the time of death could be found. Causes of death for children under GH treatment at the time of death were: respiratory infection (1.9-year-old boy), cardiac failure (14.9-year-old girl) and sudden death (2 girls nearly 12 years old). Five children were reported as never having been treated by GH, probably because they died during the first year of life prior starting GH treatment. One boy and one girl died from respiratory infection, one boy died of pneumococcal meningitis, one girl died of cardiac tamponade, and one girl suffered a sudden death.

Regarding adults who were treated with GH (*n* = 8), only 2 were under GH at the time of death: a 21-year-old woman and a 23-year-old man whose deaths were caused by functional intestinal obstruction and aneurysm, respectively.

Furthermore, 42 adults were reported to be obese in the data from the CépiDC and/or FRC-PWS database. Among them, the median age at the time of death was 32 years. The reported diabetes cases (*n* = 22; 2 17-year-old adolescents and 20 adults) were mostly associated with obesity. Causes of death among the obese adult population were respiratory failure (*n* = 23), sudden death (*n* = 8) and cardiovascular causes (*n* = 5). Infectious diseases like pneumopathy, sepsis or peritonitis accounted for 4 deaths. One patient died as a result of a refractory status epilepticus. For one patient, the cause of death was unknown.

## Discussion

Prader-Willi syndrome is a rare disorder, which explains the scarce epidemiological data. In our study, we identified 104 deaths over 11 years, which makes this one of the largest recently reported cohorts. The median age at death was 30 years, ranging from neonatal to 58 years. Seventeen deaths occurred in < 18-year-old patients, with about 70% of them occurring in ≤2-year-old children. Respiratory causes accounted for more than 50% of the deaths in patients with PWS. Among adults, most deaths were caused by respiratory failure, while respiratory infections were the primary cause of death in children.

Respiratory-related causes have been reported as the most common cause of death among children with PWS [1, 15, 21, 24]. In our child population, respiratory infections leading to death were not associated with fever in most cases. Respiratory infections without fever progressing rapidly to death have been described [14, 15], emphasizing that viral respiratory infection can progress rapidly and disproportionately in relation to the initial clinical situation. As an immune deficit has not been described in patients with PWS, this susceptibility to respiratory infection seems to be related more to a respiratory muscle hypotonia that reduces cough reflex efficiency [9, 29]. Furthermore, it may be hypothesized that silent aspirations, which particularly affect infants [30], result in a compromise of the pulmonary parenchyma that may increase the risk of respiratory infections. Of note, in 2011 and 2012 when the number of cases of death was the highest, we investigated the possibility of respiratory infection epidemics, but none was reported in France in these years.

Among the adults who died of respiratory causes, most presented restrictive ventilatory impairment, a known comorbidity in the PWS population [31]. In addition, other intrinsic features of PWS increase the risk of severe respiratory problems. Upper airway hypotonia and abnormal response to hypercapnia and hypoxia have been well-described in animal models and the PWS population [32, 33]. Comorbidities like obesity and scoliosis coupled with sleep-disordered breathing [19, 34–37] can result in exacerbations of these abnormalities and may increase the risk of death from respiratory causes. Respiratory-related deaths, as well as other causes of death, were often the result of an acute event in the context of multiple comorbidities. Unfortunately, we could not analyze the association between comorbidities and causes of death, since the comorbidities were not always listed.

In line with previous publications [14, 24, 38, 39], we found that sudden death was a major cause of death among this population in both children and adults. In the early 2000s, there were a number of reports of sudden death in children with PWS during the first year of GH treatment [20, 40–42]. In our population, among the 3 children who suddenly died under GH therapy, only 2 were on this therapy at the time of death. In both cases, the treatment had been going on for more than one year, which would make it less likely that these cases of death were related to GH use. Moreover, no substantial differences were found regarding causes of death among children with and without GH treatment. Concerning adults, no patient who suffered a sudden death was known to be under GH at the time of death. Our results are not in favor of a role of GH treatment on the deaths.

The context for sudden death was not always available. For 2 adolescents, sudden death occurred during a bath for one and while horseback riding for the other. According to the description of the entourage present at the moment of death, these patients might have experienced a malaise and collapsed under the circumstances described. Among the 14 adults who suddenly died, 5 presented respiratory comorbidity (such as chronic respiratory insufficiency or obstructive sleep apnea). Since the data on almost half the adult patients who suffered a sudden death came only from the CépiDC database, we have no information on the context for them. Moreover, a limitation of this work is that we do not have full data on all the drugs taken by these patients. A recent study by our team showed that adult patients receive multiple psychotropic treatments [43]. These treatments increase the risk of cardiac rhythm disorders [44, 45] and may lead to sudden death, a hypothesis that could not be explored in our population. Antipsychotics also aggravate alveolar hypoventilation and respiratory sleep disorders [46]. Furthermore, several cases of death were recorded as sudden death because the only information on the CépiDC database was “cardiorespiratory arrest” without further details that would have allowed the PWS expert to better classify the cause of death. This may be one reason why this was the second most common cause of death in our population.

Several previous publications, however, have identified obesity-related cardiorespiratory pathologies as the leading causes of death among adults with PWS [16, 17, 19]. In our study, deaths due to cardiac failure were classified separately from deaths to respiratory failure, limiting comparisons with the existing literature. Cardiac and vascular causes were pooled and made up the third leading cause of death in our population. Although the classifications of causes of death are not strictly identical, previous studies have already mentioned cardiac failure, probably related to obesity, as a major cause of mortality [16–18]. In our study 4 adult patients died from pulmonary thromboembolism. The risk of pulmonary embolism in this population has also been highlighted in previous years [17, 18]. In a recent American article, the proportion of pulmonary thromboembolism was 8%, while the proportion found in this study was 5% in the same age group. Besides, in the United States, cases of pulmonary thromboembolism were reported in adolescence (5% between 12 and < 18 years) that we have not observed in our population since the 4 patients who died of pulmonary thromboembolism in France were ≥ 30 years old. It is possible that we have misclassified some cases of pulmonary thromboembolism in sudden death since, as already mentioned, many cases of sudden death were identified only by the CépiDC registry. Also, even for cases identified by the FRC-PWS database, little is known about the underlying causes of sudden death because very few families agree to perform an autopsy (that it is not mandatory by law in France). For the same reasons, we cannot exclude that some cases of cardiac or respiratory failure may have been the consequence of a pulmonary embolism. This may explain the differences found with Butler’s and colleagues’ study [18]. However, pulmonary embolism is an issue to consider particularly in this population. Recently, a clinical trial was interrupted after two patients treated by a methionine aminopeptidase 2 (MetAP2) inhibitor suffered a fatal pulmonary thromboembolism [47]. Although MetAP2 inhibitors may affect vascular endothelial cell function [48], our data showed a risk of venous thromboembolic events in patients with PWS.

In contrast to the literature [18, 49], we did not find patients in our population who had died of cancer or leukemia. No cases of death due to choking were registered during the study period either. Regarding genetic subtypes, we found no differences in age of death by gender in patients with a deletion, as described in Butler’s study [18].

Some limitations should be acknowledged with regard to the interpretation of our results. First, despite our efforts to obtain as much data as possible, the genetic subtype could only be acquired for a part of our sample. Therefore, we conducted exploratory analyses to document relevant associations with genetic subtypes (causes and age of death according to the genetic subtype, differences between gender and ages in a specific genetic subtype). The results must be interpreted as such and should be confirmed by other studies involving a larger number of patients. Second, as stated, for a number of patients we only had the CépiDC data without the patient’s medical history. It is thus possible that their causes of death were misclassified. The CépiDC data depended very much on how thoroughly the death certificates had been filled out. Moreover, we were unable to determine whether the information recorded on the death certificates had been confirmed by autopsy or if the registered cause of death was secondary to an undiagnosed pathology.

Our study has several strengths. One of them is exhaustiveness at the national level. This is because we cross-checked data from two national sources of mortality (the FRC-PWS database and the CépiDc registry). On the one hand, as our results show, not all patients with PWS who died were included in the FRC-PWS database. On the other hand, 15% of the patients with PWS who died were not registered as such in the CépiDc register as this pathology would not have been included among the associated causes of death. The complementarity of these two sources of information gives us more comprehensive and robust information. Another major strength is that our analyses were based on data that encompassed the last 11 years, a period when the health of patients with PWS globally improved because of early diagnosis, multidisciplinary management, GH therapy and the implementation of the Reference Center. Today, most of the pediatric PWS population has been identified by the FRC-PWS and is followed by physicians from the PWS Competence and Reference Centers. Children are referred very early to the experts, most during the neonatal period when they are diagnosed [3]. Young people appear to be well identified and managed, reflecting good integration into the health care system. In recent years, the same improvement has been observed in adults, who nevertheless would benefit from supplementary efforts. In the upcoming years, we can assume that patients whose care has been coordinated by the Reference Center, with multidisciplinary care and GH treatment starting very early on in life, will display less obesity and fewer life-threatening comorbidities. These differences in clinical practice are likely to impact on the overall survival of PWS patients born in more recent years compared to those born from older generations. Exploring these differences requires a systematic registration of patients with PWS from birth throughout their lives. Our study design offered another approach based on the inclusion of patients with PWS who died between 2004 and 2014 regardless of their year of birth, which prevents from analyzing such trends.

Further research is needed to monitor the mortality, gather more detailed data on antecedents and contexts of death and quantify the impact of changes in clinical practice on the survival of patients with PWS. For this purpose, the FRC-PWS has developed a form for the physicians of the PWS Competence and Reference Centers to use in reporting deaths in France. This form is designed to elicit information on the cause, place and time of death, the autopsy results if performed, the genetic subtype, comorbidities, and GH treatment or other treatments. The ongoing and prospective data collection of cases of death might help to improve the power of our findings.

## Conclusion

In summary, patients with PWS die prematurely. The causes of death, particularly in adults, could be attributable to comorbidities that are frequent in this population, such as obesity. In the last two decades, numerous changes have improved the health of these patients, particularly weight management. However, the age of death remains premature and further efforts are required to prevent obesity and others comorbidities.

PWS is a life-threatening condition with an excessive risk of mortality compared to the general population. These patients present lifelong clinical fragility and premature aging. At a time when new medications for patients with PWS are being investigated, it is crucial to increase our knowledge about comorbidities, causes of death and their respective frequencies. Causes of death in this population should be considered during clinical trials to avoid misinterpretations of the relation between a death and the drug, as well as to look particularly for contraindications for a specific drug.

Last, this study investigated a significant number of deaths recorded over a limited time period, during which numerous improvements in patient care were implemented. The FRC-PWS offers a prospective opportunity for ongoing monitoring that might help to target or contraindicate some drugs and enhance knowledge of the reasons leading to death in the PWS population. Thus, the results of this study and continued monitoring might enable the implementation of preventive measures and provide advance guidance for the care of new generations of patients with PWS.

## Data Availability

The datasets generated and analyzed during the current study are not publicly available due to medical privacy but are available from the corresponding author on reasonable request.
